# Unexpected Ductility Enhancement in Crystalline–Crystalline Polyolefin Diblock Copolymers without Introducing Soft Segments

**DOI:** 10.1021/acs.macromol.5c03102

**Published:** 2026-03-03

**Authors:** Rocco Di Girolamo, Miriam Scoti, Chiara Santillo, Claudio De Rosa

**Affiliations:** Dipartimento di Scienze Chimiche, 9307 Università di Napoli Federico II, Complesso Monte S. Angelo, Via Cintia, Napoli I-80126, Italy

## Abstract

Combining polyethylene and polypropylene (isotactic or syndiotactic) crystalline blocks within a single macromolecule offers a powerful framework to elucidate how the molecular architecture governs deformation and phase transformations during stretching in polyolefins. In this study, polyethylene-*block*-isotactic-polypropylene (PE-*b*-iPP) and polyethylene-*block*-syndiotactic-polypropylene (PE-*b*-sPP) copolymers with well-defined block lengths, synthesized using single-site catalysts, were investigated to elucidate the relationship between molecular architecture, crystalline structure, and mechanical response. X-ray diffraction and tensile analyses revealed that despite the absence of amorphous soft segments, both block copolymers exhibit remarkable ductility enhancement compared to their corresponding homopolymers when a long iPP or sPP block is linked to a PE block. The mechanical performance strongly depends on the relative block lengths and the polymorphic transformations that occur during deformation. In PE-*b*-iPP samples, the α-form of iPP progressively transforms into the mesomorphic form under deformation, while in PE-*b*-sPP copolymers, the helical form I of sPP transforms into the *trans*-planar form III. These stress-induced transitions promote energy dissipation and delay fracture, enabling large deformations with pronounced strain hardening. The results demonstrate that high ductility in crystalline polyolefin block copolymers can be achieved in *hard*–*hard* systems through deformation-assisted polymorphic transitions, offering an alternative molecular design strategy without introducing soft segments for tough, extensible crystalline materials.

## Graphical abstract

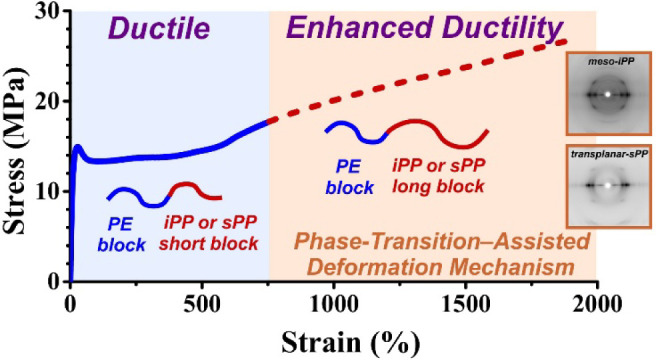

## Introduction

Crystalline polyolefins play a fundamental role in a wide range of engineering and industrial applications due to their tunable mechanical performance, chemical resistance, and processability.^1^ Among these materials, the two most extensively produced globally, polyethylene (PE) and polypropylene (PP), are semicrystalline thermoplastics, with their mechanical behaviors closely linked to their molecular structure and crystalline arrangement.^2,3^ Despite their fossil origin, there is still considerable interest in developing these materials, both because of their outstanding performances and because efficient chemical and mechanical recycling processes have now been successfully developed to recover them.^4–6^ In particular, in polypropylene-based materials, a key factor is the stereoregularity of the polymer chains, as the isotacticity and syndiotacticity significantly influence the level of crystallinity, polymorphism, morphology, and the resulting mechanical behavior.^7,8^ Several investigations have established clear correlations between chain microstructure, especially defects in stereoregularity and regioregularity, the resulting crystal structure, and the material’s physical properties.^9,10^

In addition to acting on tacticity, the mechanical properties of these materials can be finely tuned by incorporating comonomers into the polymer backbone, which reduces crystallinity and modifies tensile strength and impact resistance.^11–14^ Alternatively, advanced molecular design and physical blending strategies can also be employed to achieve precise control over their mechanical behavior.^15^

Among the various molecular design strategies, the mechanical performance of polyolefins can also be modified by introducing block architecture such as combining segments with different mechanical or crystallization properties to tune toughness, elasticity, or other desired physical characteristics.^16–19^

For example, efficient thermoplastic elastomer applications based on polyolefin block copolymers with high melting domains (*hard* blocks) from polypropylene or polyethylene that alternate with *soft* segments presenting low glass transition temperatures, typically amorphous random ethylene–propylene copolymers, can be nowadays obtained using specific catalytic systems that can polymerize olefin monomers in a living or controlled fashion to produce diblock or triblock copolymers where the properties of highly crystalline blocks, such as polyethylene (PE) and syndiotactic or isotactic polypropylene (sPP, iPP), can be efficiently tuned by introducing one or more soft blocks.^20–25^

This concept has been efficiently applied by Dow Chemical Company that developed olefin block copolymers^26^ (OBC) made from ethylene and a higher α-olefin using chain shuttling technology, developing materials with a statistical multiblock architecture, composed by *soft* and amorphous blocks (ethylene–octene copolymer) that alternate with *hard* and crystalline polyethylene blocks incorporating a very low amount of 1-octene comonomeric units. With this approach, more than one chain per catalyst site is formed, making it industrially relevant.

The effects of block architecture on the structure, morphology, and, in particular, on the mechanical properties of OBC polyolefin-based systems containing a crystalline hard block linked to a second (amorphous or slightly crystalline) *soft* block, as well as well-defined di- and triblock copolymers or multiblock architectures, prepared via coordinative chain transfer polymerization, have been extensively documented in the literature.^27–31^ Factors such as the length of the *soft* and *hard* blocks, their relative content, as well as the number of blocks have been shown to play a significant role in determining these properties.^32^

A more intriguing and less explored case is represented by *hard*–*hard* diblock copolymers constituted by a polyethylene block chemically linked to a stereoregular polypropylene (syndiotactic or isotactic) block.^33–35^ The mutual effect of two crystalline blocks on the mechanical behavior is, indeed, poorly documented^36,37^ in the current literature, but its study could offer the possibility to elucidate how molecular architecture governs deformation and phase transformations in crystalline block copolymers (BCP).

In the past decade, thanks to the development of living metallorganic single-site catalysts, crystalline–crystalline block copolymers with well-defined architecture and controlled molecular weight and polydispersity such as poly­(ethylene)-*block-*isotactic-polypropylene (PE-*b*-iPP) or poly­(ethylene)-*block*-syndiotactic-polypropylene (PE-*b*-sPP) have been efficiently synthesized.^19,34^ Their complex crystallization behavior,^38–40^ the phase separation,^41,42^ compatibilization mechanism in PE/iPP blends,^33–35,37^ and the possibility to tune the morphology in order to obtain oriented microstructures via epitaxial crystallization^42,43^ have been reported in literature and in some of our previous studies.

In this paper, the mechanical properties of these crystalline block copolymers, namely, focusing on block copolymers based on polyethylene linked to isotactic or syndiotactic polypropylene (PE-*b*-iPP, PE-*b-*sPP) have been investigated. This study pays special attention to how modifications at the molecular level, like block architectures and the different crystallization behavior, in both cases of isotactic or syndiotactic configurations affect their structure–property relationships, allowing to tune strength and ductility. The study highlights the intricate connections between molecular configuration and mechanical performance associated with phase transformations occurring during stretching, providing valuable insights for the thoughtful design of high-performance polymer materials and proving that the enhancement in ductility is not exclusively attributed to the presence of a *soft* or amorphous block in the molecular architecture of the BCPs.

## Experimental Section

Samples of PE-*b*-iPP have been synthesized using the Hf-pyridylamido complex activated with B­(C_6_F_5_)_3_ (Chart S1A), PE-*b*-sPP samples have been prepared using a fluorinated bis­(phenoxyimine) titanium dichloride complex (Chart S1B) activated with methylalumoxane (see Supporting Information). The values of molecular mass, polydispersity, weight fractions of blocks, and melting temperature (*T*_m_) of all the BCP samples, PE, iPP, and sPP homopolymers, are reported in Table 1.

**Table 1:** Total Molecular Mass (*M*_n_), Polydispersities (*M*_w_/*M*_n_), Molecular Masses of PE, iPP, and sPP Blocks, Weight Fractions (*w*) of Each Block, and Melting Temperature (*T*_m_) of All the BCP Samples

Sample	*M*_n_ ^a^ (g/mol)	*M*_w_ */M*_n_ ^a^	*M*_n(PE)_ ^b^ (g/mol)	*M*_n(iPP)_ ^a^ (g/mol)	*M*_n(sPP)_ ^a^ (g/mol)	*w*_PE_ ^c^ (wt %)	*w*_iPP_ ^c^ (wt %)	*w*_sPP_ ^c^ (wt %)	*T*_m_(II)^d^ (°C)
PE^e^	122400	1.30	-	-	-	100	-	-	135
iPP^f^	139850	1.29	-	-	-	-	100	-	135
sPP^e^	185000	1.27	-	-	185000	-	-	100	143
PE-*b*-iPP-1	163500	1.19	98900	64600	-	48	52	-	136
PE-b-iPP-2	180600	1.26	86000	94600	-	36	64	-	132
PE-*b*-sPP-1	151100	1.26	80600	-	70500	49	-	51	133
PE-*b*-sPP-2	135600	1.29	52600	-	83000	35	-	65	131

a From GPC.

b Determined from the total molecular mass *M*_n_ and the molecular mass of the iPP or sPP block as *M*_n(PE)_ = *M*_n_ – *M*_n(iPP or sPP)_.

c Evaluated from ^13^C NMR spectra (Figure S1).

d Determined from DSC second heating scan collected at 10 °C/min (Figures S2–S3).

e Synthesized with the Ti catalyst reported in Chart S1B.

f Synthesized with the Hf catalyst reported in Chart S1A.

All samples, including the polyethylene (PE) homopolymer used as a reference, are characterized by a molecular weight (*M*_n_) higher than 120000 g/mol and polydispersity (*M*_w_/*M*_n_) below 1.3.

Mechanical properties were evaluated on compression-molded films prepared by slow cooling from the melt. Measurements were performed using a universal testing system (Zwick/Roell BTC-FR2.5TH.D09) in accordance with the ASTM D882 standard for thin plastic films. During the tests, the drawing rate-to-initial-length ratio was set to 10 mm (mm × min)^−1^ for recording stress–strain curves, and to 0.1 mm (mm × min)^−1^ for determining Young’s modulus. Each stress–strain curve and corresponding mechanical parameter represents an average of at least five independent measurements.

Experimental details of the characterization by DSC and X-ray powder and fiber diffraction are also described in the Supporting Information.

## Results and Discussion

### Structural and Thermal Behavior

X-ray powder diffraction profiles of compression-molded samples of PE-*b*-iPP, PE-*b*-sPP with different block lengths, and of the PE homopolymer crystallized by cooling the melt (≈15 °C/min) to room temperature are reported in Figure 1, whereas X-ray profiles of the sPP and iPP homopolymers are reported in Figure S4.

**Figure 1:**
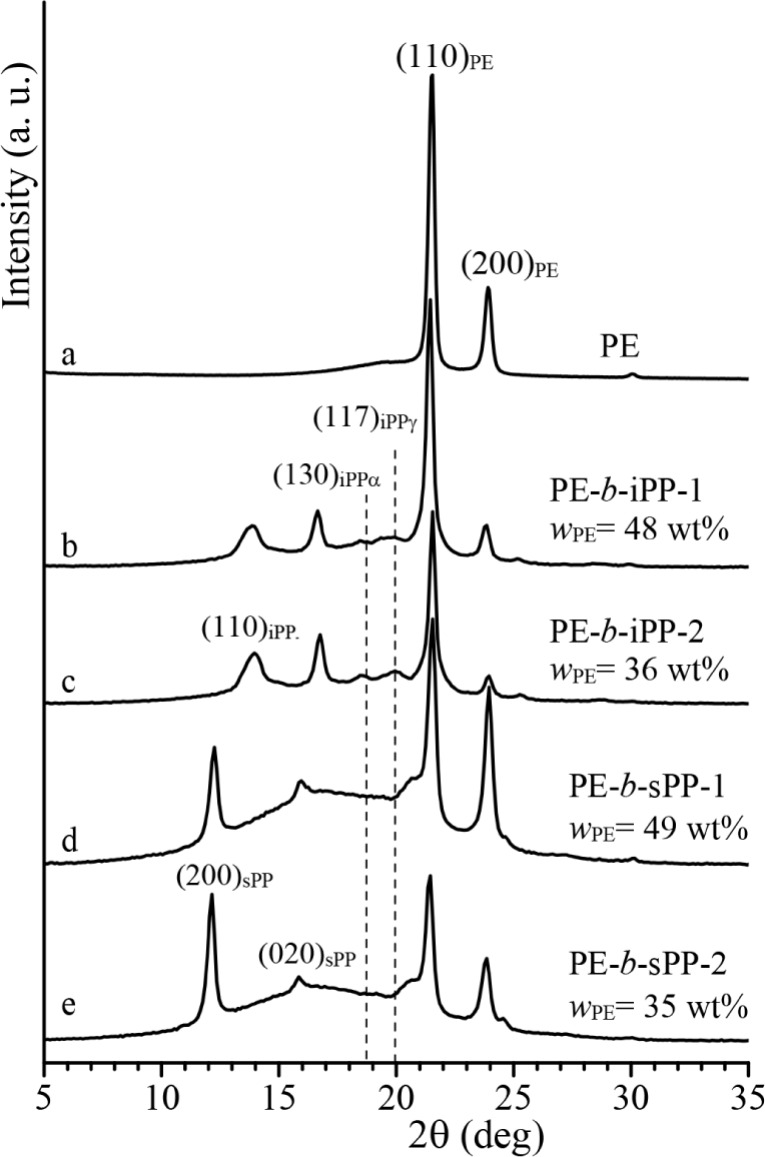
X-ray powder diffraction profiles of compression-molded samples of PE-*b*-iPP and PE-*b*-sPP BCPs and of the PE homopolymer. The (130)_α_ and (117)_γ_ reflections at 2θ = 18.6° and 20.0° of the α- and γ-form of iPP, respectively; the (110)_PE_ and (200)_PE_ reflections at 2θ = 21.4° and 23.9°, respectively, of the orthorhombic form of PE; and the (200)_sPP_ and (020)_sPP_ reflections of form I of sPP at 2θ = 12.2° and 16°, respectively, are indicated. The weight fraction of the PE block (*w*_PE_) is also reported.

It is apparent that the iPP block in the PE-*b*-iPP copolymers crystallizes in the α-form with a small fraction of crystals in the γ-form, as indicated by the presence in the diffraction profiles b and c of Figure 1 of the (110)_α_ and (130)_α_ reflections at 2θ ≈ 14° and 18.6°, respectively, of the α-form, and the (117)_γ_ reflection of low intensity of the γ-form at 2θ = 20°. Moreover, the (110)_PE_ and (200)_PE_ reflections at 2θ = 21.4° and 23.9°, respectively, of the orthorhombic form of PE are also observed (profiles b and c of Figure 1), indicating that both iPP and PE blocks are crystalline. The relative intensity of the (110)_PE_ and (200)_PE_ reflections of PE increases with increasing PE weight fraction in the BCP, with the overall crystallinity ranging from 55 to 60%.

The diffraction profiles of samples PE-*b*-sPP-1 and PE-*b*-sPP-2 (profiles d and e in Figure 1) indicate the presence of a double crystalline phase (sPP and PE) also in the PE-*b*-sPP copolymers. This is evidenced by the sharp 200 and 020 reflections of form I of sPP at 2θ = 12° and 16°, together with the (110)_PE_ and (200)_PE_ reflections of the orthorhombic form of PE at 2θ = 21.4° and 24°. Crystallization from the melt of the BCPs, therefore, produces the development of different crystalline forms, in both PE-*b*-iPP and PE-*b*-sPP samples that mutually interact during crystallization,^38–40^ through a reciprocal nucleating effect with an impact on their morphology and mechanical response.

Samples of PE-*b*-iPP copolymers exhibit in the DSC curves a single broad peak centered at 132–136 °C in both the heating and cooling scans (Figure S2). This behavior arises from the overlap of the iPP and PE melting and crystallization processes, consistent with the moderate isotacticity of the iPP block obtained by using the living Hf catalyst. A similar behavior is observed for PE-*b*-sPP copolymers, which, despite containing two crystalline phases, display only one melting endotherm (Table 1, Figure S3).

### Mechanical Behavior

The mechanical stress–strain curves of the melt-crystallized samples of PE-*b*-iPP and PE-*b*-sPP BCPs with different block lengths (Table 1) and of the PE homopolymer reported as a reference are shown in Figures 2 and 3, respectively.

**Figure 2:**
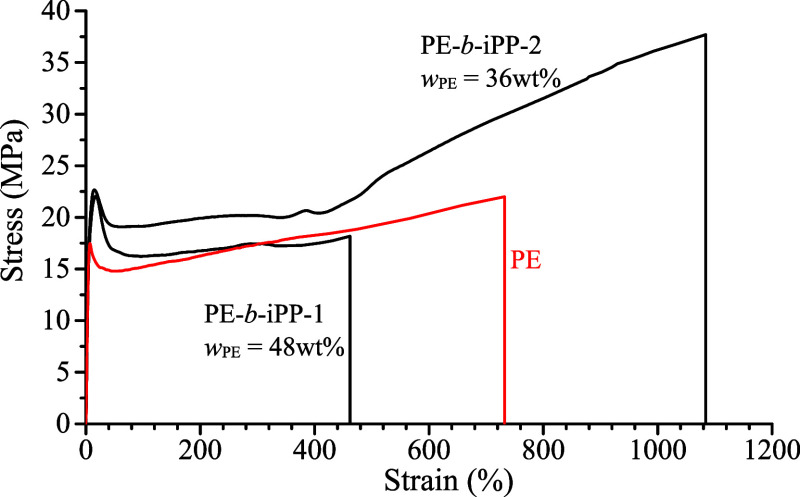
Stress–strain curves of the melt-crystallized compression-molded films of PE-*b*-iPP copolymers and of the PE homopolymer.

**Figure 3:**
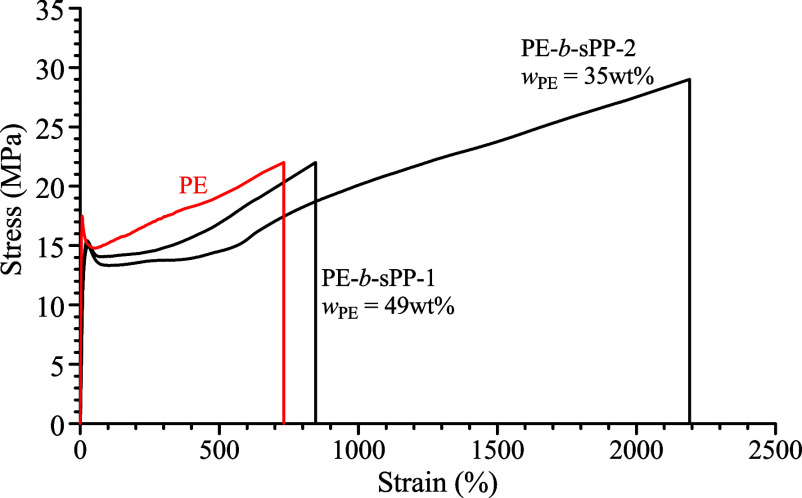
Stress–strain curves of the melt-crystallized compression-molded films of PE-*b*-sPP copolymers and of the PE homopolymer.

The PE homopolymer exhibits characteristics of thermoplastic behavior with evident yielding, a strain-softening region, and limited strain hardening up to the breaking point at about 730% strain, with a value of the Young’s modulus of ∼500 MPa (Table 2). The addition of an iPP block to a polyethylene segment (samples PE-*b*-iPP-1 and PE-*b*-iPP-2) has the effect to modify the mechanical behavior depending on the PE/iPP relative block length. For both samples, PE-*b*-iPP-1 and PE-*b*-iPP-2, the presence of a second hard block (iPP) leads to an increase in the yield stress (reaching 22–23 MPa compared to 18 MPa for the PE homopolymer), a moderate effect on the Young’s modulus and a significant influence on the ductility and the stress at break (Table 2). In both cases, the increase in yield stress compared to PE, approaching the value of the iPP homopolymer (approximately 20 MPa), is clearly due to the presence of the second crystalline block of iPP. The presence of a longer iPP block (sample PE-*b*-iPP2, *w*_*PE*_ = 35%) produces a relevant improvement in ductility and a contemporaneous increase in overall strength, with values of deformation at break of 1080% with evident strain-hardening that allows achieving high stress at break of 38 MPa. In the sample PE-*b*-iPP-1, the presence of a short iPP block linked to the PE block produces a material with deformation at break lower than that observed for the PE homopolymer. The relatively short iPP block likely does not provide sufficient crystalline continuity or load-bearing capability to effectively transfer stress from amorphous regions to the crystalline lamellae during stretching. Consequently, the material cannot sustain extensive chain alignment and the associated deformation mechanisms assisted by complete phase transitions that typically promote ductility, leading to premature failure and reduced elongation at break.

**Table 2:** Elastic Modulus (*E*), Stress (*σ_y_*), and Strain (*ε_y_*) at the Yield Point, Stress (*σ*_b_) and Strain (*ε*_b_) at Break, Tension Set (*t*_b_), Evaluated from the Stress–Strain Curves of Figures 2 and 3, and Crystallinity (*x*_c_), Evaluated from the X-ray Diffraction Profiles of Figure 1, of Melt-Crystallized Compression-Molded Samples of PE-*b*-iPP and PE-*b*-PP BCP Slowly Cooled from the Melt

Sample	*E* (MPa)	*σ*_y_ (MPa)	*ε*_y_ (%)	*σ*_b_ (MPa)	*ε*_b_ (%)	*t*_b_ (%)	*x*_c_ (%)
PE	460 ± 10	18 ± 2	7 ± 1	22 ± 2	730 ± 12	-	65
iPP	420 ± 10	20 ± 2	10 ± 2	25 ± 2	750 ± 120	470 ± 50	50
sPP	200 ± 10	16 ± 2	15 ± 2	16 ± 3	390 ± 100	180 ± 20	55
PE-*b*-iPP-1	517 ± 15	22 ± 2	18 ± 1	18 ± 1	460 ± 130	490 ± 30	60
PE-*b*-iPP-2	353 ± 8	23 ± 2	16 ± 1	38 ± 2	1080 ± 150	484 ± 40	55
PE-*b*-sPP-1	160 ± 7	15 ± 1	25 ± 2	22 ± 2	850 ± 160	288 ± 60	38
PE-*b*-sPP-2	151 ± 14	15 ± 1	29 ± 4	29 ± 2	2190 ± 170	335 ± 20	41

This suggests that a different deformation mechanism acts in the two block copolymers depending on the PE/iPP relative block length.

It is worth highlighting that both block copolymers PE-*b*-iPP-1 and PE-*b*-iPP-2 present a similar degree of crystallinity, ranging from 55 to 60%, a factor that cannot, by itself, explain the observed differences in mechanical behavior.

A comparison of the mechanical behavior of the sample PE-*b*-iPP-2 with those of the iPP and PE homopolymers (Figure 4) clearly indicates that the block copolymer exhibits a remarkable enhancement in ductility and higher stress values at all deformation levels compared to the corresponding homopolymers, confirming that tuning the block length allows to obtain a good strength–ductility balance.

**Figure 4:**
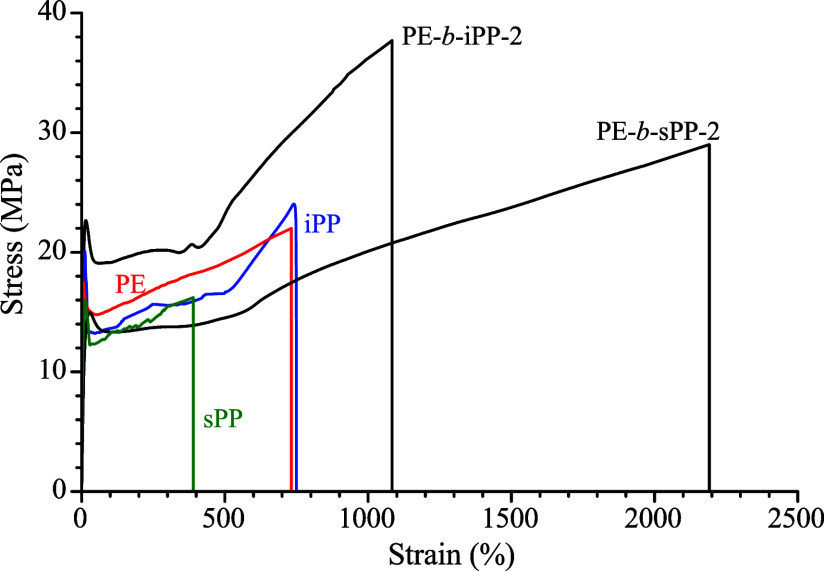
Stress–strain curves of the two most ductile samples of block copolymers (PE*-b*-iPP-2 and PE-*b*-sPP-2) compared with those of PE, iPP, and sPP homopolymers (Table 1).

It is worth noting that the ductile behavior of the iPP homopolymer, obtained using the same Hf-based living catalyst employed for the synthesis of the BCPs and shown in Figure 4, is associated with the moderate isotacticity imparted by the Hf catalyst (*mmmm* = 92%), in contrast to the higher isotacticity typically achieved for iPP synthesized via heterogeneous Ziegler–Natta catalysis that is usually associated with higher crystallinity and brittle mechanical behavior.

These data show that an increase in ductility can be obtained not only in *hard*–*soft* block copolymer, where a crystalline block is linked to a rubbery amorphous block, but also in *hard–hard* block copolymers, where the presence of two crystalline phases and differences in their amounts (block lengths) play a crucial role in the deformation mechanism. The chain architecture dictates a different mechanism of deformation and/or fracture that is strictly connected to the phase transformations that occur during stretching as demonstrated in the next section.

A similar behavior is observed in the case of PE-*b-*sPP BCPs whose stress–strain curves are reported in Figure 3 in comparison with that of the PE homopolymer. Both samples PE-*b*-sPP-1 and PE-*b*-sPP-2 present good ductility, values of the Young’s modulus of about 150–160 MPa, and yield stress of 15 MPa (Table 2) in agreement with the value of crystallinity of ∼40%, lower than that observed for the PE homopolymer (65%). However, also in the case of PE-*b*-sPP samples, a strong effect on the values of ductility can be observed depending on the PE/sPP block length. When a longer sPP block is linked to the PE segment, a significant gain in ductility is observed, as in the case of PE-*b*-iPP samples previously discussed. While the sample PE-*b*-sPP-1 with a similar block length presents a moderate ductility, similar to that of the PE homopolymer or sPP homopolymer with similar tacticity,^7^ in the case of the sample PE-*b*-sPP-2 a strong enhancement in ductility is observed with values of deformation at break of 2000%, that is, more than twice what was observed for the sample PE-*b*-sPP-1. Moreover, the sample PE-*b*-sPP-2 shows strain hardening and a high value of stress at break (29 MPa). Given the comparable degree of crystallinity in both samples, this intriguing mechanical behavior, similar to that observed in PE-*b*-iPP copolymers, relies on the molecular architecture as well as the mechanisms of deformation and fracture, which are significantly influenced by the length of the propylene blocks (either iso or syndio). In principle, the different molecular architecture and block lengths can affect the crystal morphology and explain the different mechanical behavior.

Small Angle X-ray Scattering and Transmission Electron Microscopy (SAXS and TEM) studies of similar samples indicate that these samples crystallize from the melt via a breaking out mechanism^41,42^ and develop an intricate lamellar morphology, where the lamellar crystals of PE and iPP or sPP alternate with amorphous layers and each crystalline layer is sandwiched by its own amorphous phase (PE, iPP, or sPP),^38,40^ in agreement with crystallization from a homogeneous melt or weakly segregated melt. The absence of a well-defined spherulitic morphology (Figure S5) obtained from this complex crystallization scenario is in agreement with the degree of crystallinity and the values of the Young’s modulus observed for these samples (Table 2).

These results demonstrate that by tuning the block length of the isotactic (iPP) and/or syndiotactic polypropylene (sPP) segments linked to a polyethylene block, both high strength and ductility can be achieved resulting in mechanical performance comparable to, or even exceeding, in terms of tensile strength, that of crystalline–amorphous block copolymers.^26,44^

### Phase Transformations during Stretching

A study of the structural transformations occurring during tensile deformation has been performed for both the series of PE-*b*-iPP and PE-*b*-sPP copolymers to clarify the role of different crystalline phases in their mechanical behavior.

The X-ray fiber diffraction patterns of the four samples of PE-*b*-iPP and PE-*b*-sPP BCPs obtained by stretching at room temperature compression-molded films of Figure 1 at different values of strain ε are reported in Figures 5–8.

**Figure 5:**
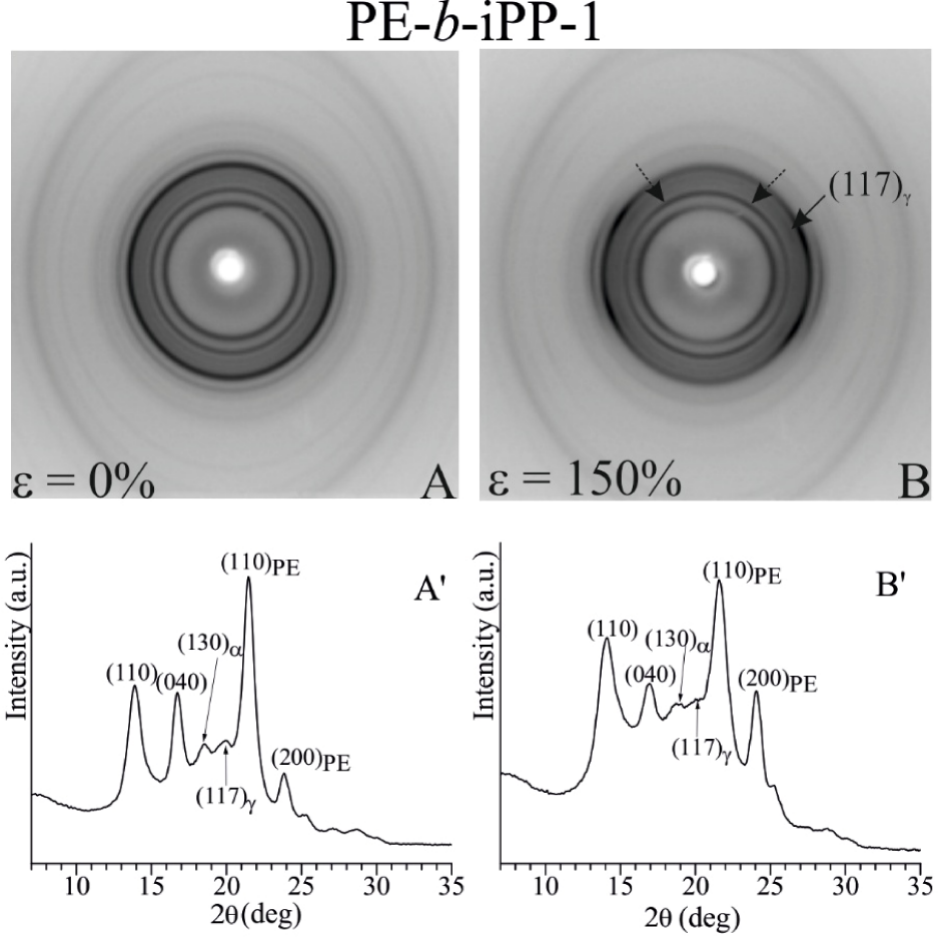
X-ray diffraction patterns (A–B) and intensity profiles read along the equator (A′–B′), of the sample PE-*b*-iPP-1 with 48 wt % PE, before stretching (A) and after stretching compression-molded films at 150% strain at room temperature (B). The dashed arrows in part B indicate the off-equatorial polarization of the (040)_α_ reflection of the α-form and the (008)_γ_ reflection of the γ-form.

As already shown in Figure 1, the X-ray diffraction pattern of the initial melt-crystallized unstretched film of the sample PE-*b*-iPP-1 (Figure 5 A,A′) show the presence of the (130)_α_ reflection of the α-form and the low intensity (117)_γ_ reflection of the γ-form of iPP and (110)_PE_ and (200)_PE_ reflections at 2θ = 21.4° and 23.9° of PE. This indicates that the sample crystallizes basically in the α-form of iPP with a small portion of crystals in the γ-form and in the orthorhombic form of PE. However, the low intensity of both the (130)_α_ and (117)_γ_ reflections may indicate crystallization in a disordered modification intermediate between the α- and γ-form.

During deformation, a general orientation of crystals of both iPP and PE blocks occurs. At a low strain of 150% (Figure 5B), a partial polarization of the (110)_PE_ reflection of PE at 2θ = 21° on a layer line off the equator is observed, indicating a tilted orientation of PE crystals with chain axes tilted with respect to the stretching direction.

The iPP crystalline phase also assumes a tilted orientation with the chain axes of crystals of α- and γ-form oriented nearly normal to the stretching direction (cross-β or perpendicular *c*-axis orientation)^45,46^ (Figure 5B). This is indicated by the polarization of the reflection at 2θ ≈ 17°, corresponding to the (040)_α_ reflection of the α-form and/or the (008)_γ_ reflection of the γ-form, in a nearly meridional position on a layer line off the equator, and the polarization of the reflection at 2θ ≈ 20°, corresponding to the (117)_γ_ reflection of the γ-form, on a layer line off the equator (Figure 5B).

A slight polarization of the (111)_α_ reflection at 2θ ≈ 21° of the α-form on the first layer line (Figure 5B) reveals that some of the crystals of the α-form are also aligned with the chain axes parallel to the stretching directions.

Generally, in iPP homopolymer the cross-β orientation of crystals of γ-forms, with chain axes oriented normal to the stretching direction, transforms into the standard fiber orientation by stretching at higher degrees of deformation accompanied by the transformation of the γ-form into the α-form or in the mesomorphic form of iPP.^45–48^ Due to the limited ductility of the sample PE-*b*-iPP-1 (Figure 2), it was not possible to record X-ray fiber diffraction patterns of this sample at high strains and detect any eventual phase transformation and/or alignment of crystals in the standard fiber orientation.

In the case of the more ductile sample PE-*b*-iPP-2 with a longer iPP block, the diffraction patterns have been recorded during stretching up to much higher values of strains and reveal the occurrence of phase transformations at high deformations (Figure 6). As already shown in Figure 1, the sample PE-*b*-iPP-2 is crystallized in the undeformed state in a mixture of crystals of α- and γ-form of iPP and the orthorhombic form of PE (Figure 6 A,A′).

**Figure 6:**
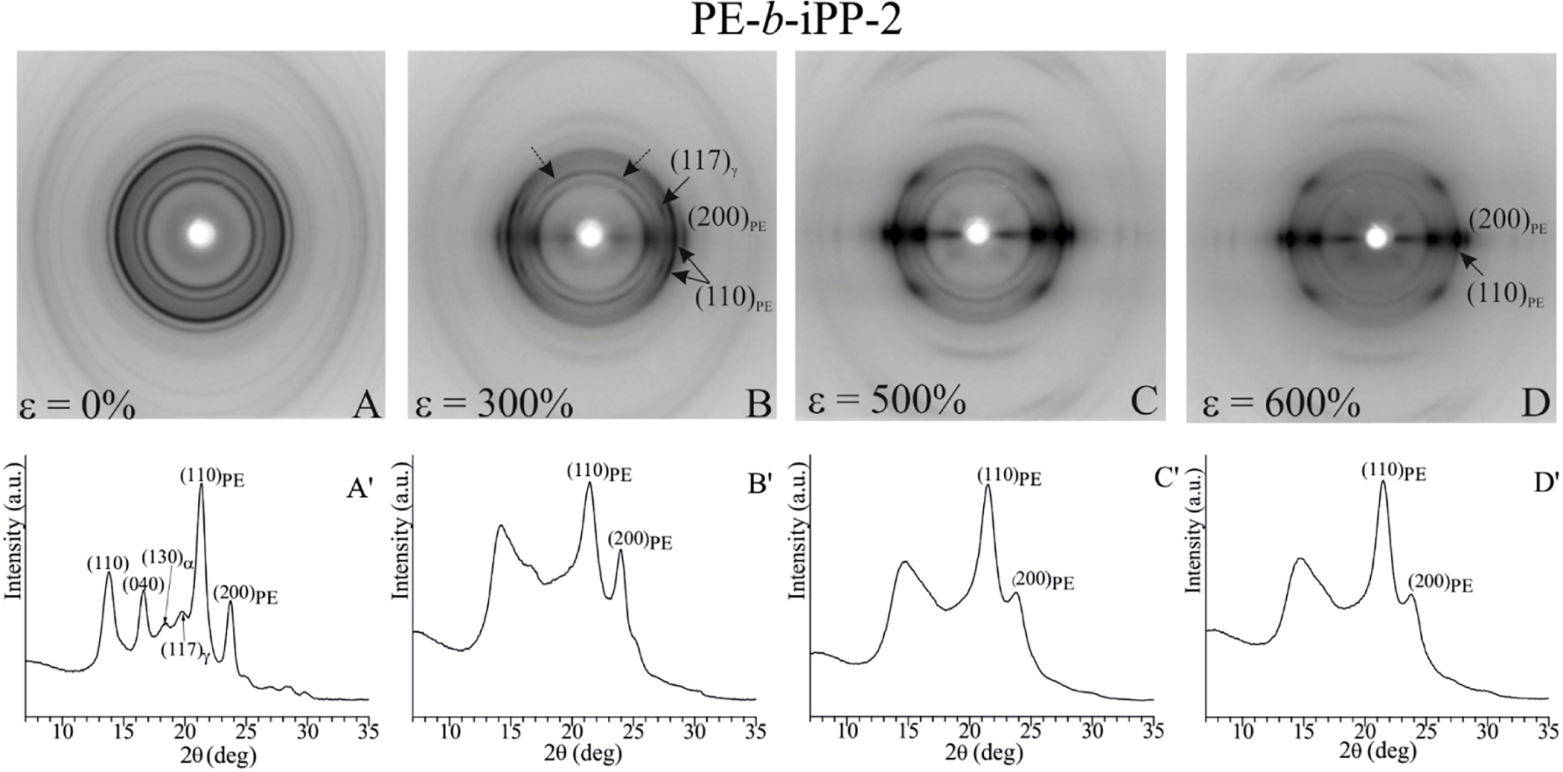
X-ray fiber diffraction patterns (A–D) and intensity profiles read along the equator (A′- D′), of the sample PE-*b*-iPP-2 with 36 wt % PE, before stretching (A) and after stretching compression-molded films at room temperature at the indicated different degrees of deformation ε (B–D). The dashed arrows in B indicate the off-equatorial polarization of the (040)_α_ reflection of the α-form and the (008)_γ_ reflection of the γ-form.

At a deformation of 300%, a portion of the crystals of the iPP α-form transforms into the mesomorphic form of iPP (Figure 6B,B′).^47^ In fact, the equatorial (110)_α_, (040)_α_, and (130)_α_ reflections of the α-form present in the pattern of Figure 6A are replaced in the diffraction pattern of Figure 6B by a broad equatorial halo centered at 2θ ≈ 15°, typical of the mesomorphic form.^48^ However, the diffraction pattern of Figure 6B shows that the (117)_γ_ reflection of the γ-form at 2θ ≈ 20° is still present and strongly polarized on a layer line off the equator, indicating the presence of crystals of the γ-form or in α/γ disordered modifications closer to the γ-form not yet transformed into the mesomorphic form oriented with chain axes nearly perpendicular to the stretching direction (cross-β orientation). This is also clearly demonstrated by the presence in the pattern of Figure 6B of a weak (008)_γ_ reflection of the γ-form at 2θ ≈ 17° polarized on the meridian. This indicates that at low degrees of deformation (300%), while crystals of the α-form of iPP rapidly transform into the mesomorphic form, crystals of the γ-form or in α/γ modifications closer to the γ-form are oriented with the chain axes normal to the stretching direction and do not yet transform into the mesomorphic form.

At a higher degree of deformation (500–600%), also the crystals of the γ-form transform into the mesomorphic form with a high orientation of the chain axes, aligned only parallel to the stretching direction (Figure 6C–D).

Analogously, at low deformations, crystals of the orthorhombic form of PE are initially oriented with the chain axes tilted with respect to the stretching direction, as proved by the slight polarization of the (110)_PE_ reflection of PE at 2θ = 21° on the layer line off the equator in the diffraction pattern of Figure 6B. At higher degrees of deformation, crystals of PE are aligned as in a standard fiber orientation with chain axes parallel to the stretching direction, as demonstrated by the strong polarization of the (110)_PE_ and (200)_PE_ reflections of PE on the equator in the diffraction pattern of Figure 6C–D.

In summary, the diffraction pattern of Figure 6 indicates that in the sample PE-*b*-iPP-2 with a longer iPP block, both crystals of the α and γ-forms of iPP transform into the mesomorphic form, but the α-form transforms more easily than the γ-form at lower deformations. This transformation occurs through the formation at low strains of cross-β structures characterized by crystals of γ-form not yet transformed into the mesomorphic form oriented with chain axes nearly perpendicular to the stretching direction. Crystals of the orthorhombic form of PE are also oriented at low strains with chain axes tilted with respect to the stretching direction before achieving a standard fiber orientation at high strains. At a high degree of deformation, well-oriented fibers with mixtures of crystals of the mesomorphic form of iPP and of the orthorhombic form of PE, with chain axes aligned along the stretching direction are obtained (Figure 6D).

The transformation during stretching of the α-form into the mesomorphic form provides a mechanism for the conversion of mechanical energy that favors stress transfer and retards sample failure. This mechanism of deformation assisted by phase transitions during stretching has been well documented for iPP samples with different microstructures prepared with metallocene catalysts and for Ziegler–Natta iPP samples crystallized under different conditions.^45,47^ In our case of crystalline–crystalline BCPs, where the iPP block is linked to a PE block, this deformation mechanism assisted by phase transitions plays a clear role in the sample PE-*b*-iPP-2 with a longer iPP block (64 wt %). However, the whole mechanical behavior is also defined in these BCP samples by the morphology considering that these samples crystallize from a microphase-separated melt via a breaking out mechanism in a disordered spherulitic morphology where PE and iPP crystalline lamellae are intimately interconnected and alternate within the macro-spherulitic morphology.^40,42^

The bidimensional X-ray diffraction patterns of the PE-*b*-sPP BCPs, reported in Figures 7–8, confirm, as already shown in Figure 1 (profiles d, e), that the unstretched specimens of samples PE-*b*-sPP-1 and PE-*b*-sPP-2 are mainly crystallized in mixtures of crystals of the orthorhombic form of PE and form I of sPP. In particular, in the unoriented specimen PE-*b*-sPP-1 with similar block lengths, the sPP block crystallizes mainly in helical form I with a minor amount of form II, as revealed by the presence in the diffraction pattern of Figure 7A,A′ of the strong 200 and 020 reflections at 2θ = 12.2° and 16, respectively, of form I of sPP and the shoulder at 2θ = 17.8° corresponding to the 110 reflection of form II of sPP.^7^ The PE block is crystallized in the orthorhombic form, as proven by the presence of the (110)_PE_ and (200)_PE_ reflections at 2θ = 21.4° and 23.8°, respectively (Figure 7A).

**Figure 7:**
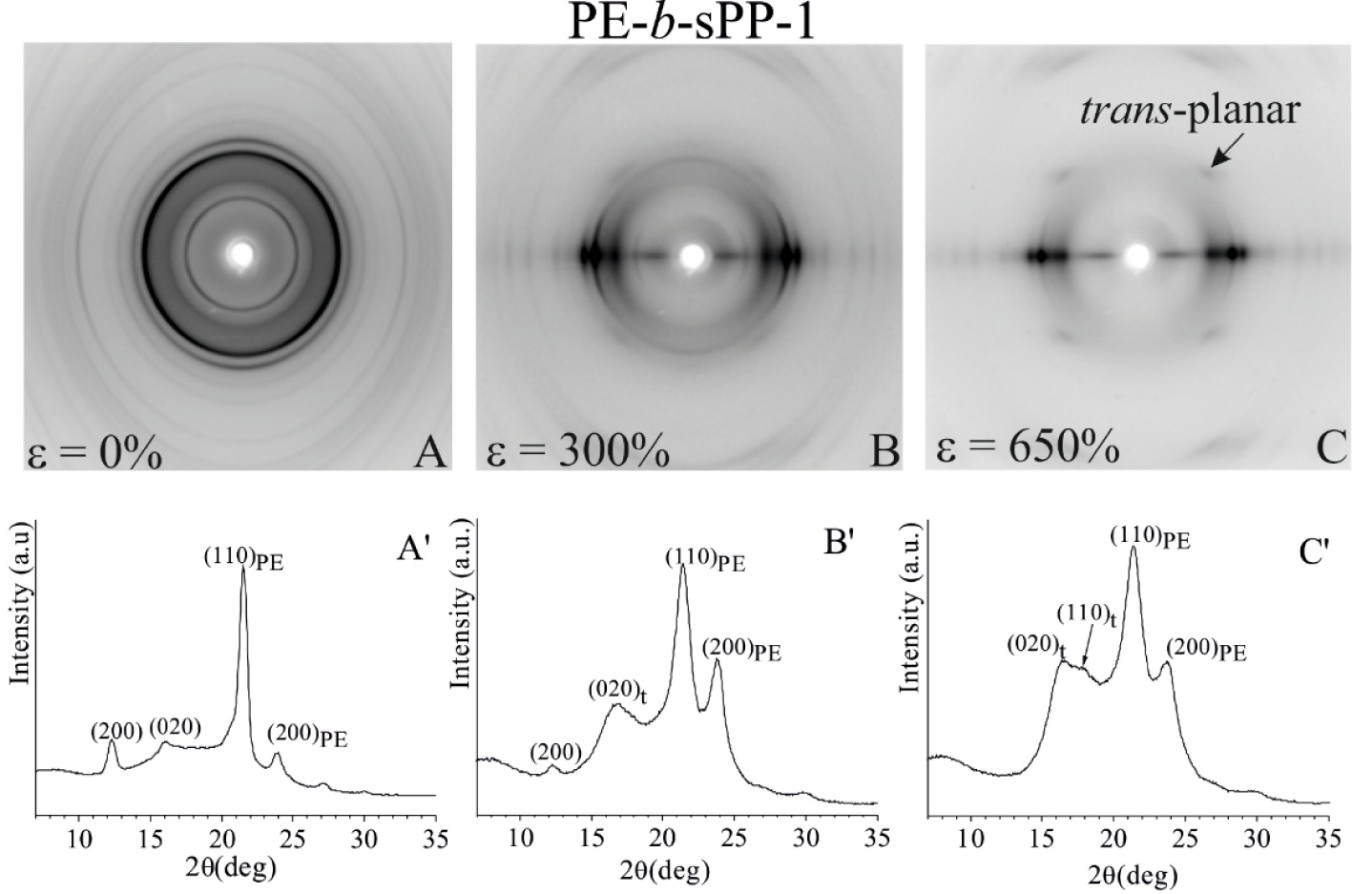
X-ray fiber diffraction patterns (A–C) and intensity profiles read along the equator (A′–C′) of the sample PE-*b*-sPP-1 with 49 wt % PE, before stretching (A) and after stretching compression-molded films at room temperature at values of strain ε of 300% (B) and 650% (C).

**Figure 8:**
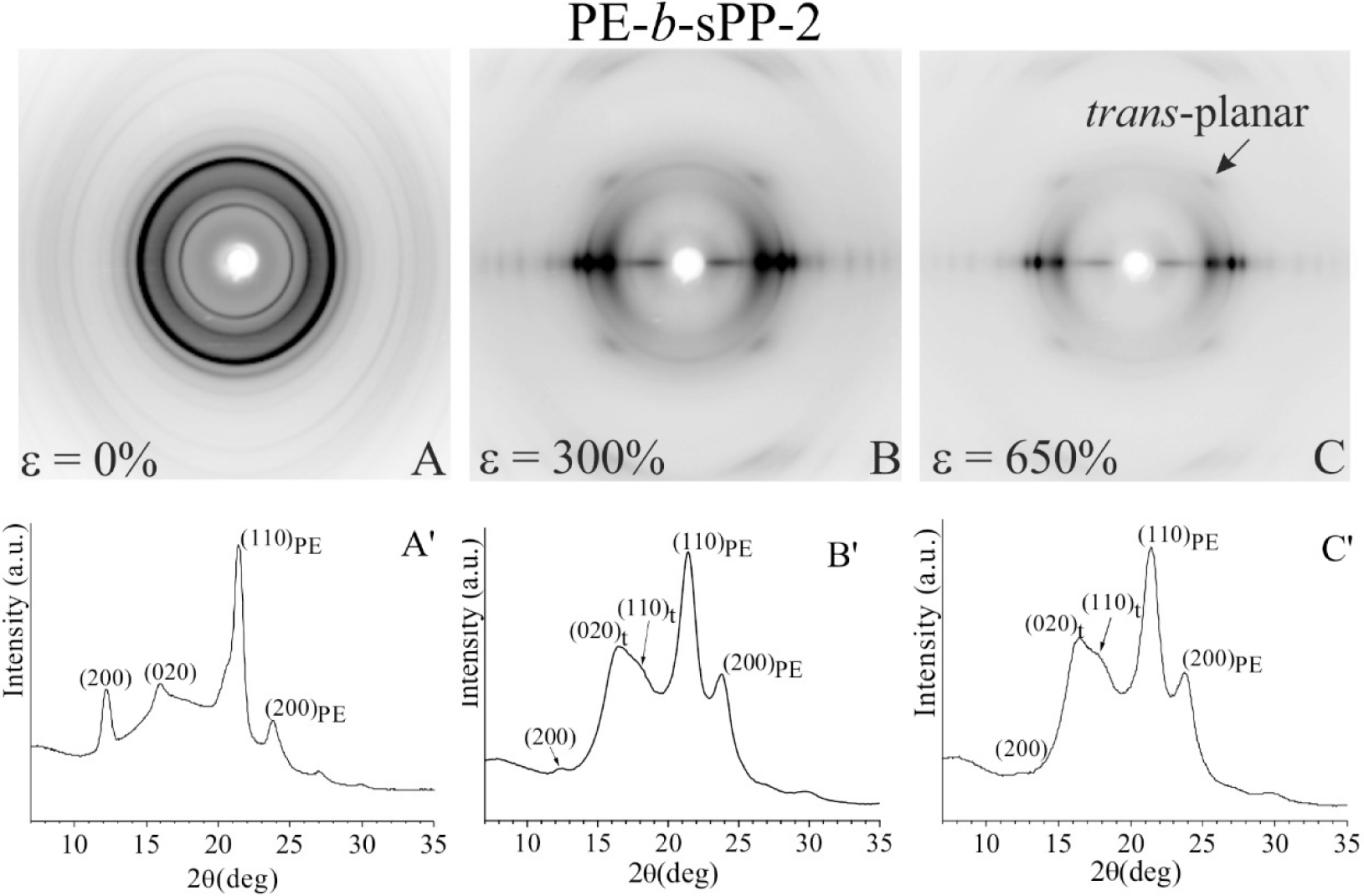
X-ray fiber diffraction patterns (A–C) and intensity profiles read along the equator (A′–C′) of the sample PE-*b*-sPP-2 with 35 wt % PE, before stretching (A) and after stretching compression-molded films at room temperature at values of strain ε of 300% (B) and 650% (C).

The X-ray diffraction pattern of the sample PE-*b*-sPP-1 stretched at moderate deformation (ε = 300%) in Figure 7B shows that the form I of sPP with chains in 2/1 helical conformation begins transforming into the form III of sPP with chains in *trans*-planar conformation,^7^ as evidenced by a drop in the intensity of the (200) reflection at 2θ = 12.2° of form I and the emergence of (020)_t_ and (110)_t_ reflections at 2θ = 16.4° and 17.9°, characteristic of form III, and of the first-layer reflection associated with a *trans*-planar periodicity of 5.1 Å. The PE orthorhombic phase displays an increasing crystal orientation, and no new polymorphic forms appear under these conditions.

As deformation progresses further (Figure 7C), the relative amount of *trans*-planar form III of sPP increases, and the orientation of both sPP and PE crystals with chain axes aligned along the stretching direction increases. This is clearly proven in the diffraction pattern of Figure 7C, where the (200) reflection of form I at 2θ = 12.2° has almost disappeared, while the intensity of the equatorial (020)_t_ and (110)_t_ reflections at 2θ = 16.4° and 17.9° of form III and of the first-layer reflection associated with the *trans*-planar periodicity of 5.1 Å increases, along with the increase in polarization of the (110)_PE_ and (200)_PE_ reflections of PE on the equator.

A similar behavior has been observed for the sample PE-*b*-sPP-2 with a longer sPP block. Also in this sample, the sPP form I present in the unoriented sample (Figure 8A,A′) progressively transforms into *trans*-planar form III during stretching, and crystals of the orthorhombic form of PE only orient with chain axes parallel to the stretching direction (Figure 8 B–C,B′–C′). At high deformation (650%), a very low amount of form I is still present, evidenced by the presence of the low-intensity (200) reflection at 2θ = 12.2° in Figure 8C that potentially could transform into form III at higher deformation,^7^ and well-oriented fibers with mixtures of crystals of the *trans*-planar form III of sPP and of the orthorhombic form of PE with chain axes aligned along the stretching direction are obtained (Figure 8C). The higher sPP weight fraction in PE-*b*-sPP-2, compared to PE-*b*-sPP-1, together with its ability to undergo stress-induced phase transitions and its more disordered crystalline morphology (see Figure S5), results in enhanced ductility.

These data confirm that in PE-*b*-iPP and PE-*b*-sPP BCPs, the presence of a higher fraction of a polypropylene crystalline phase (iPP or sPP) linked to PE, capable of undergoing phase transitions during stretching, provides an unexpected improvement in ductility, delaying failure. In fact, while an improvement in mechanical properties would have been expected by attaching amorphous or soft blocks to a crystalline PE block giving a *hard–soft* architecture, against all expectations the designed molecular architecture with two linked crystalline *hard* blocks (*hard–hard* architecture) produces a great enhancement of ductility. However, although these materials can undergo very high deformations, the presence of a crystalline block capable of phase transitions during stretching cannot fully compensate for the advantages provided by soft amorphous blocks with low glass transition temperatures but gives further advantage of high modulus and strength. We have demonstrated that by controlling the molecular architecture and polymorphic behavior, it is possible to enhance ductility while simultaneously maintaining high values of Young’s modulus and stress over the entire deformation strain range.

## Conclusions

This study shows that *crystalline*–*crystalline* block copolymers made of PE linked to iPP or sPP blocks (PE-*b*-iPP, PE-*b*-sPP) can achieve relevant ductility improvements, even without any *soft*, amorphous segments. The mechanical properties of these materials rely on the lengths of the blocks and on the crystallization behavior of iPP or sPP. In both cases, increasing the polypropylene block fraction significantly boosts elongation at break and strain hardening even when the overall crystallinity remains similar.

X-ray diffraction analyses during deformation indicated that this enhanced ductility arises from stress-induced polymorphic transitions within the polypropylene domains: the transformation of the α-form into the mesomorphic form in iPP and the transition of the helical form I into the *trans-*planar form III in sPP. These phase changes, along with gradual crystal reorientation, serve as an effective way to accommodate stress and dissipate energy, which helps delay fractures and allows for larger deformations and increased toughness. This mechanism is unexpectedly enhanced when a high amount (longer blocks) of iPP or sPP crystalline phase is present. These transformations, along with the block architecture and the disordered spherulitic morphology of alternated PE/iPP or sPP lamellae, facilitate the extensibility of these materials.

In summary, these findings highlight that ductility can be enhanced in polyolefin block copolymers, preserving strength, through crystalline–crystalline (*hard–hard*) structures, without needing amorphous soft blocks if crystalline phases capable of undergoing transitions during stretching are present. The relationship between block length, crystalline phase behavior, and transformations induced by deformation offers a robust design strategy for customizing the mechanical response of fully crystalline polyolefin materials for applications that require both strength and flexibility.

## Supplementary Material



## Notes

Experimental procedures and additional polymer characterization data (PDF)

## #

Institute of Polymers, Composites and Biomaterials-CNR, 80055 Portici, Italy
