# Supplementary material for: Unexpected Ductility Enhancement in Crystalline–Crystalline Polyolefin Diblock Copolymers without Introducing Soft Segments
Source: Macromolecules. 2026 Mar 3;59(6):3672–81. doi: 10.1021/acs.macromol.5c03102 (PMC13019973; doi:10.1021/acs.macromol.5c03102)
Supplement: Supplementary file 1 [file ma5c03102_si_001.pdf]

Supporting Information for the paper:

Unexpected Ductility Enhancement in Crystalline-Crystalline Polyolefin Di-Block Copolymers Without Introducing Soft Segments

Rocco Di Girolamo, Miriam Scoti, Chiara Santillo,*[†] *Claudio De Rosa**

Dipartimento di Scienze Chimiche, Università di Napoli Federico II, Complesso Monte S. Angelo, Via Cintia, I-80126 Napoli, Italy.

[†] Current Address: Institute of Polymers, Composites and Biomaterials-CNR, 80055 Portici, Italy

Experimental Details

Synthetic Procedure of block copolymers. Samples of diblock copolymers constituted by PE and iPP blocks (PE-*b*-iPP) of different lengths, and samples of corresponding PE and iPP homopolymer were prepared as described with the catalyst based on pyridylamidohafnium complex activated with $B(C_6F_5)_3$ (Chart S1,A) The polymerizations were conducted at 22 °C in toluene (150 mL) in a reactor charged with a mixture of the pyridylamidohafnium catalyst (30 μmol) and 1 equivalent of cocatalyst $B(C_6F_5)_3$. The co-polymerizations were carried out sequentially by first charging propylene (2–3 g) and then, after complete consumption of propylene (about 20 min reaction time), charging ethylene a pressure of 2–3 atm (about 10 min reaction time). Upon completion of the reaction and precipitation of the block copolymer sample, the reactor was vented and acidic MeOH (5% HCl, 10 mL) was injected. The polymer sample was recovered by vacuum filtration and dried under vacuum at 60 °C. Samples of PE-*b*-sPP copolymers and PE homopolymer were prepared with a living organometallic catalyst, phenoximine–titanium complex (Chart S1,B) (~10mg), activated with dried

methylalumoxane (d-MAO) (~10mg). The co-polymerizations were carried out sequentially by first charging ethylene (15 PSI for 30 min) and then, after complete consumption of ethylene (about 10 min reaction time), charging propylene at pressure of 40 PSI (about 50 min reaction time). Upon completion of the reaction and precipitation of the block copolymer sample, the reactor was vented and acidic MeOH (5% HCl, 10 mL) was injected. The polymer sample was recovered by vacuum filtration and dried under vacuum at 60 °C.

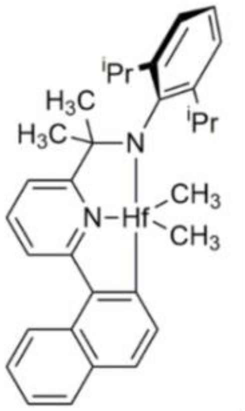

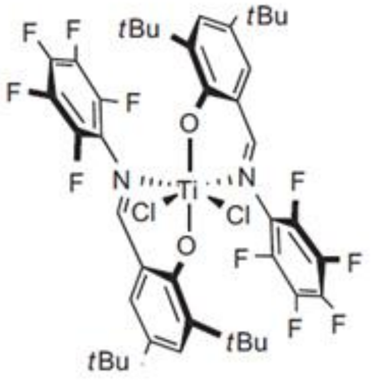

A **B**

Chart S1. Hafnium (A) and Titanium (B) complexes used as catalysts for the preparation of the BCP samples. Catalyst A was also used to synthesize the iPP homopolymer and catalyst B for the PE homopolymer.

^{13}C NMR Characterization. The microstructures of all copolymer samples-have been studied with ^{13}C NMR spectroscopy. All spectra were obtained using a Bruker DPX-400 spectrometer operating in the Fourier transform mode at 120 °C at 100.61 MHz. The samples were dissolved with 8% wt/v concentration in 1,1,2,2-tetrachloroethane-d$_2$ at 120 °C. The carbon spectra were acquired with a 90° pulse and 15 seconds of delay between pulses and CPD (WALTZ 16) to remove ^{1}H-^{13}C coupling. About 1500-3000 transients were stored in 32K data points using a spectral window of 6000 Hz. ^{13}C NMR spectra of all samples of the PE-*b*-iPP and PE-*b*-sPP copolymers are reported in Figure S1.

X-ray diffraction and DSC. X-ray diffraction patterns have been recorded at room temperature with Ni filtered Cu Kα radiation (wavelength λ=0.15418 nm). The powder diffraction profiles have been obtained with an automatic PANalytical Empyrean diffractometer operating in the reflection geometry with continuous scans of the 2θ angle and scanning rate of 0.02 degree/s, whereas the fiber diffraction patterns were recorded on a BAS-MS imaging plate (FUJIFILM) using a cylindrical camera and digitized with a digital imaging reader (Perkin Elmer Cyclone Plus).

Thermal analysis (DSC) The calorimetric measurements were performed with a Mettler-DSC822 operating in N_2 atmosphere. The sample, typically 5 mg, was placed in a sealed aluminum pan, and the measurement was carried out from -40 to 180°C using a heating and cooling rate of 10 °C min^{-1}.

GPC Analysis. The molecular mass and the polydispersity of the samples were determined by gel permeation chromatography (GPC), using an Agilent PL-220 equipped with a RI detector and three Agilent PL-Gel Mixed B columns and one PL-Gel Mixed B guard column on polymer solutions in 1,2,4-trichlorobenzene at 1.0 mL/min at 150 °C vs polyethylene standards.

Degree of crystallinity. The values of the degree of crystallinity (x_c) were determined from the X-ray powder diffraction profiles as the ratio $x_c = (I_c/I_t)$ between the intensity of the diffraction of the crystalline phase I_c and the total diffraction intensity of the semicrystalline sample $I_t = I_c + I_a$, where I_a is the scattering intensity of the amorphous phase. The diffraction intensity of the crystalline phase I_c is generally evaluated as the area of the Bragg peaks A_c observed in the X-ray powder diffraction profiles, whereas the total diffraction intensity corresponds to the area of the whole diffraction profile A_t after subtracting the background intensity, generally defined as a baseline. The degree of crystallinity is, therefore, evaluated as $x_c = (A_c/A_t)\times100$ with $A_t = A_c + A_a$, where A_a is the area of the scattering halo of the amorphous phase. The determination of the area of the diffraction of the crystalline phase A_c as the area of the Bragg peaks is related to the determination of the scattering halo of the amorphous phase. In fact the area of the Bragg peaks A_c is generally evaluated by subtracting

the area of the scattering halo of the amorphous phase A_a from the area of the whole diffraction profiles A_t.

^{13}C NMR spectra of block copolymers

PE-*b*-iPP-1 A

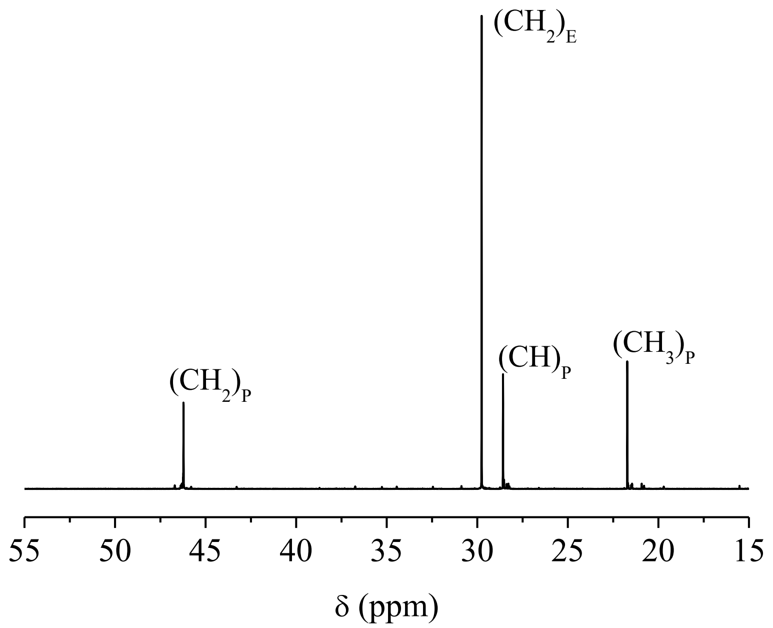

PE-*b*-iPP-2 B

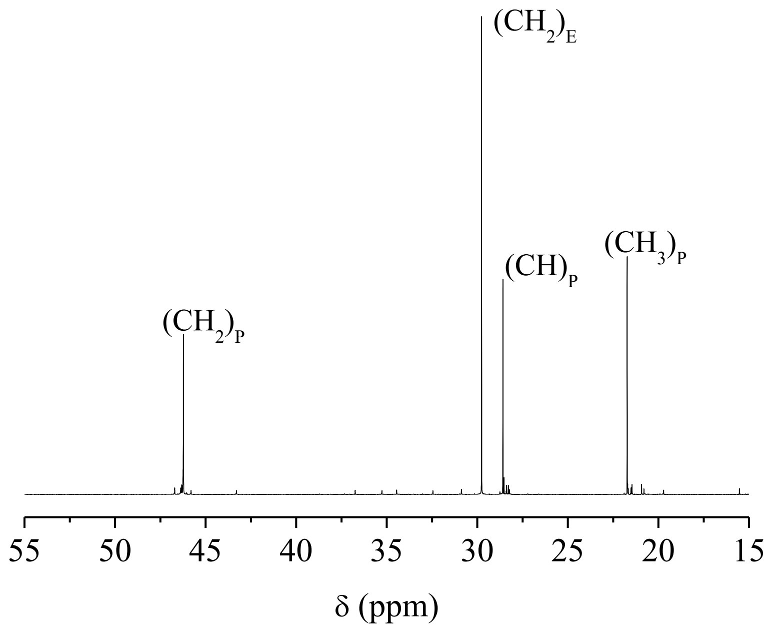

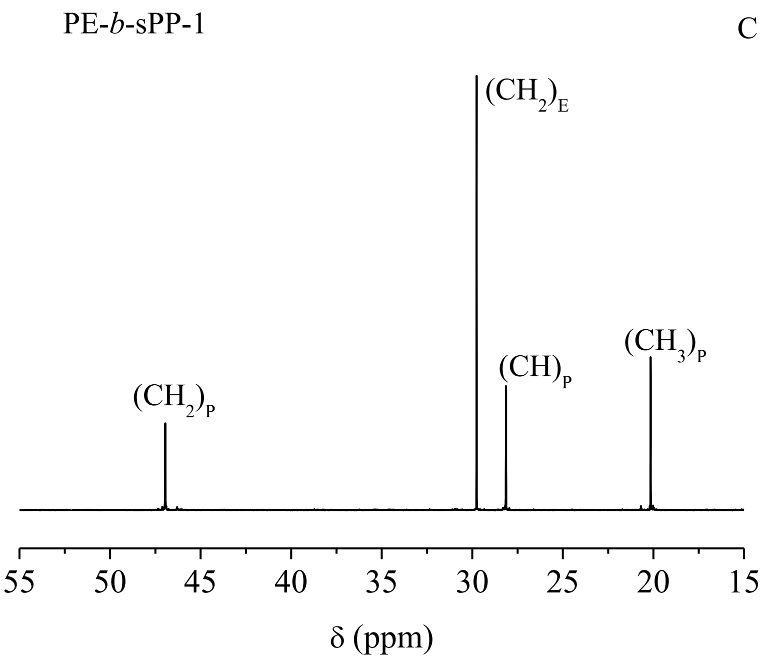

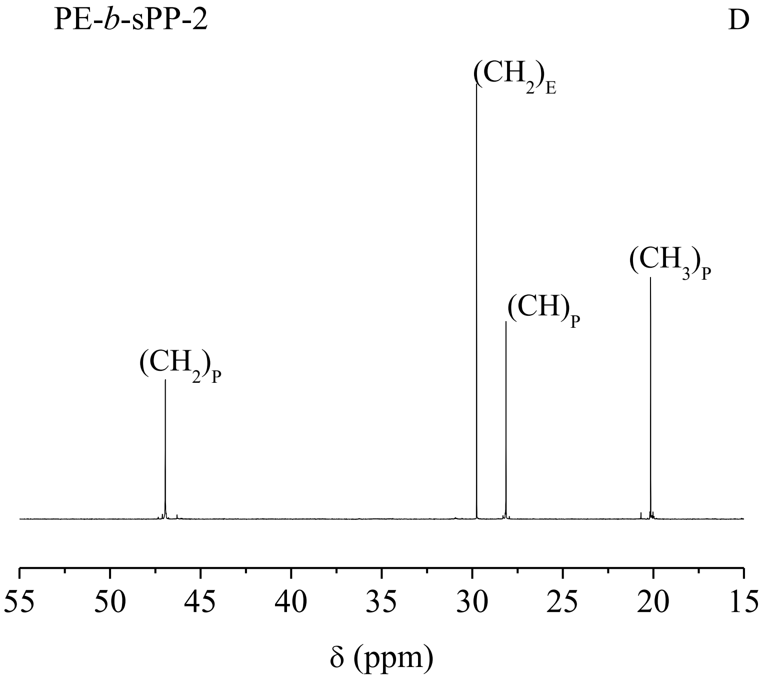

Figure S1. ^{13}C NMR spectra of PE-*b*-iPP (A, B) and of PE-*b*-sPP (C, D) samples.

Thermal analysis (DSC) data

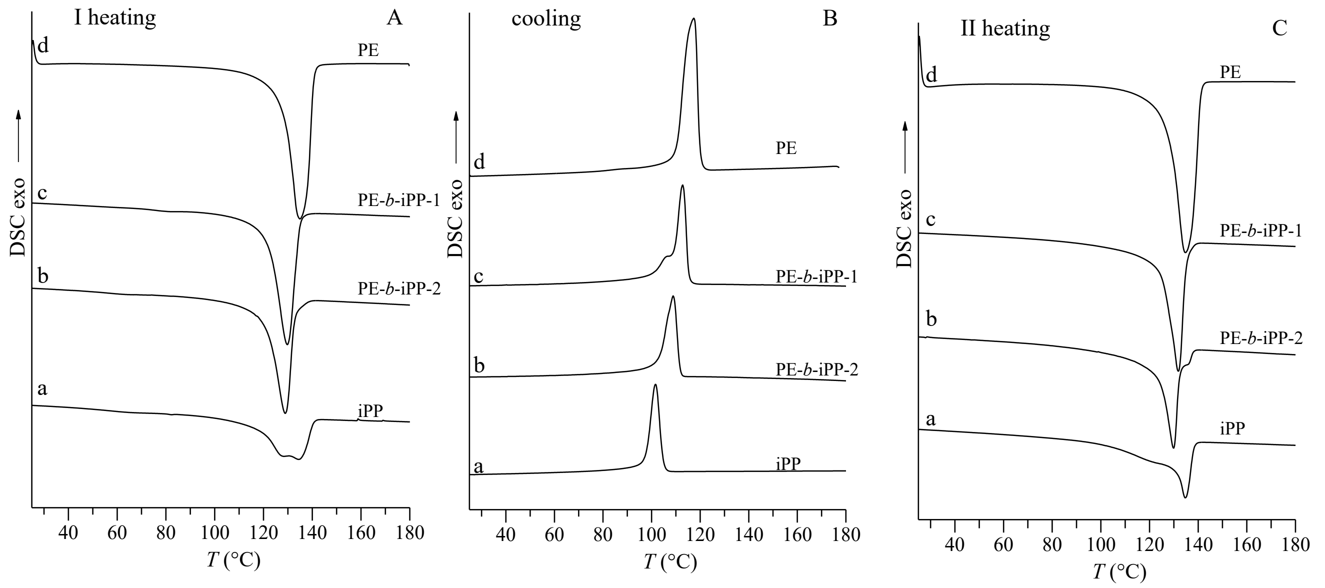

Figure S2. DSC curves of PE-*b*-iPP copolymers and PE and iPP homopolymers recorded at 10 °C/min during heating of as-polymerised samples (A), cooling from the melt (B) and successive heating (C).

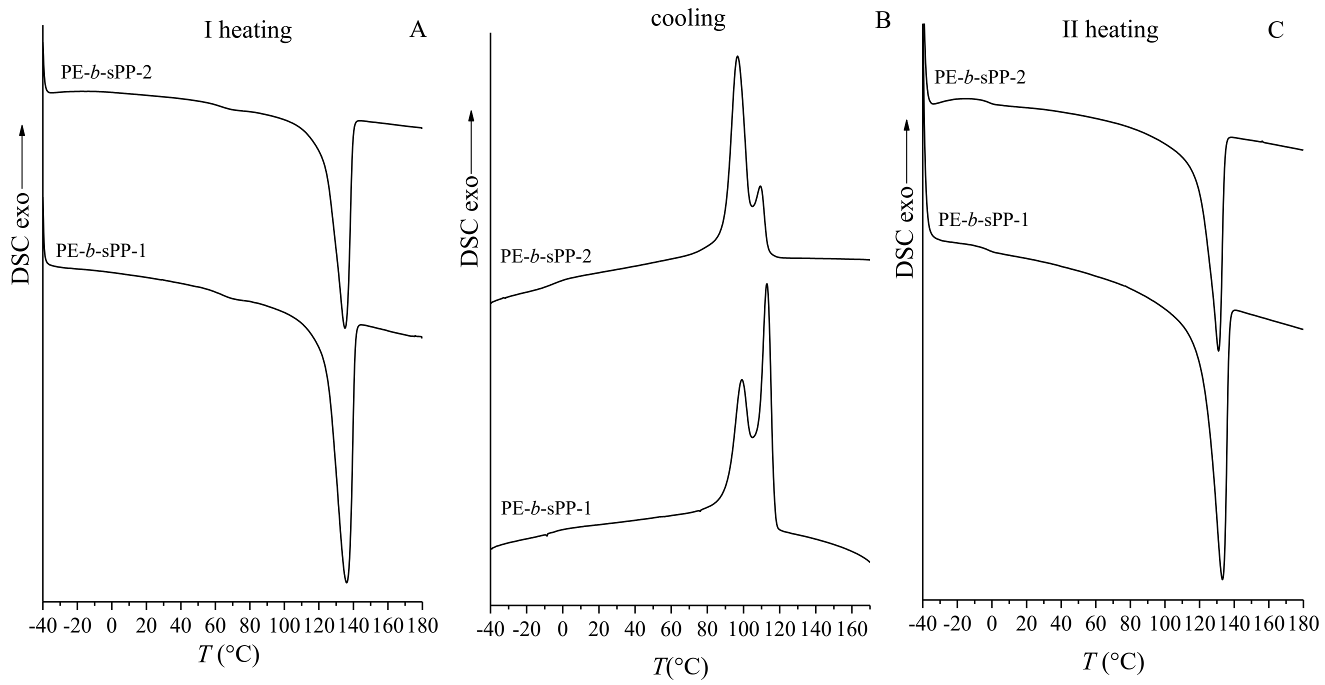

Figure S3. DSC curves of PE-*b*-sPP copolymers recorded at 10 °C/min during heating of as-polymerised samples (A), cooling from the melt (B) and successive heating (C).

Supplementary X-ray diffraction profiles.

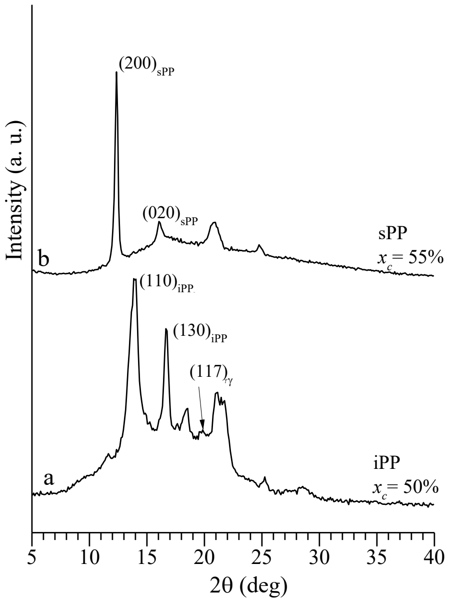

Figure S4. X-ray powder diffraction profiles of compression molded samples of iPP and sPP homopolymers. The degree of crystallinity (x_c) is indicated.

Polarized Optical Microscopy (POM) Images

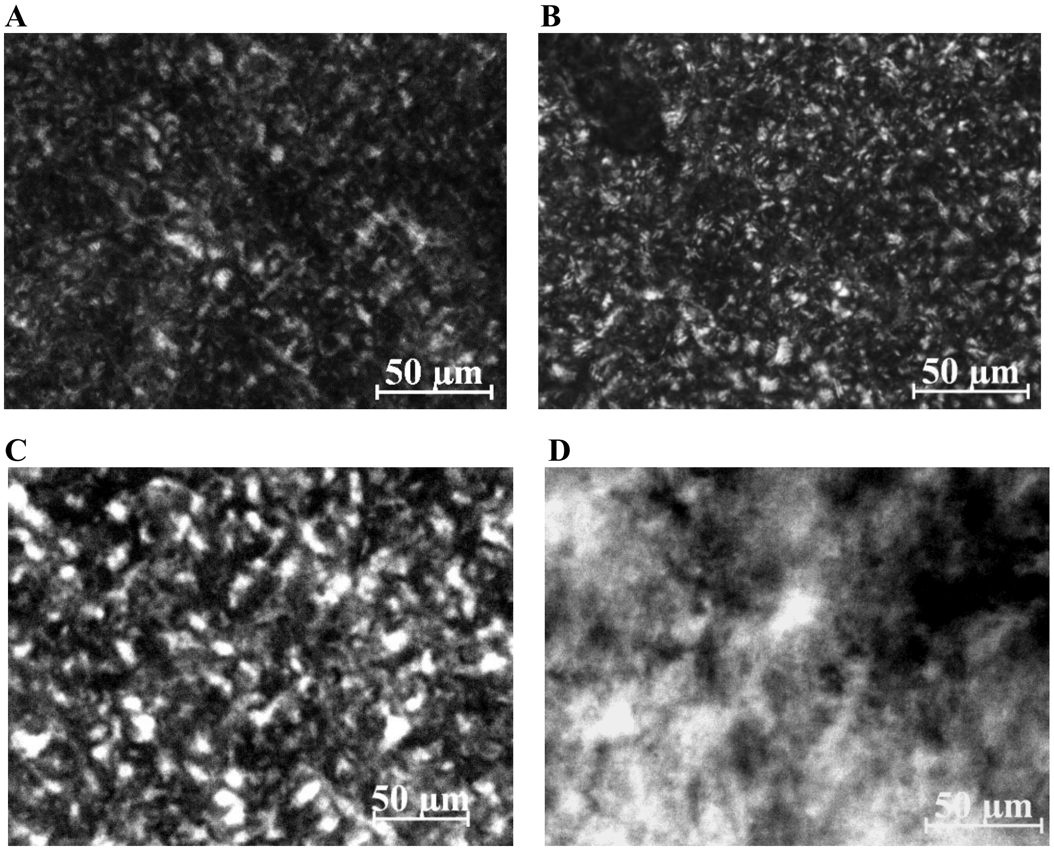

Figure S6. Polarized optical microscope images (crossed polars) recorded at room temperature of PE-*b*-iPP-1(A) and PE-*b*-iPP-2 (B) copolymers and of PE-*b*-sPP-1(C) and PE-*b*-sPP-2 (D) samples, crystallized from the melt by cooling to room temperature at cooling rate of 10 °C/min. The thickness of the films is ≈ 100 µm.

Additional X-ray fiber diffraction patterns

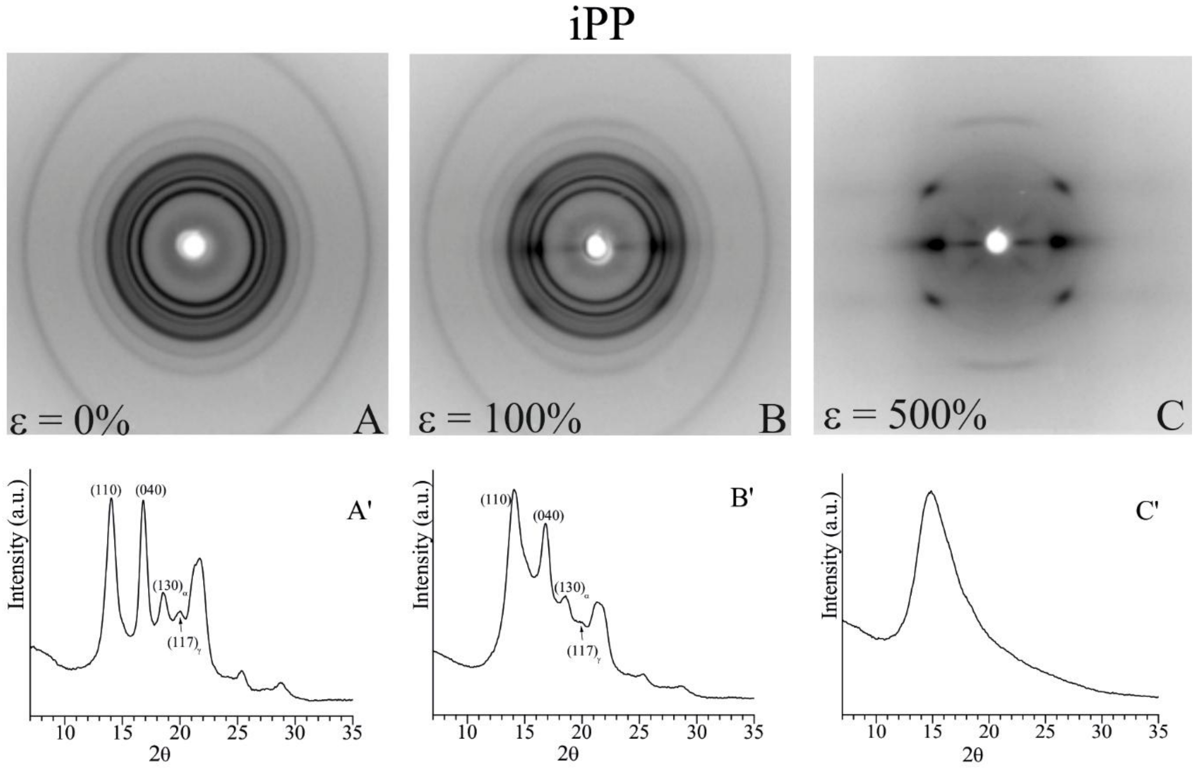

Figure S7. X-ray fiber diffraction patterns (A-C), and corresponding equatorial profiles (A'- C'), of the iPP homopolymer unoriented (A) and of oriented fibers obtained by stretching at room-temperature compression molded films at values of strain ε of 100% (B) and 500% (C).
